# Health-related quality of life among Djiboutian women with gynecological cancers, predominantly cervical cancer : a cross-sectional analytical study

**DOI:** 10.1186/s12905-026-04755-x

**Published:** 2026-07-31

**Authors:** Zam-Zam Moussa Ali, Amal Boutib, Kaiss Saleh Said, EL Madani Saad, Abderraouf Hilali, Nadira Abdallah Yahya, Abdelghafour Marfak, Ibtissam Youlyouz-Marfak

**Affiliations:** 1 Laboratory of Health Sciences and Technologies, Higher Institute of Health Sciences, Hassan First University of Settat, Settat, Morocco; 2 Higher Institute of Health Sciences, Djibouti, République de Djibouti; 3 Euro-Mediterranean University of Fès (UEMF), Fez, Morocco; 4 National School of Public Health, Ministry of Health and Social Protection, Rabat, Morocco

**Keywords:** Gynecological cancers, Health-related quality of life, SF-36, Djibouti, Physical health, Supportive care

## Abstract

**Background:**

Gynecological cancers represent a major public health problem in resource-limited settings, where diagnostic delays and limited access to oncology care can worsen clinical outcomes. In Djibouti, data on health-related quality of life (HRQoL) among women with gynecological cancers remain scarce. This study aimed to assess HRQoL using the SF-36 and to explore factors associated with physical and mental scores.

**Methods:**

A cross-sectional analytical study with prospective data collection was conducted between February and April 2024 in two referral centers in Djibouti. Ninety women with histologically confirmed cervical, endometrial, or ovarian cancer (predominantly cervical cancer) were consecutively included. HRQoL was assessed using the validated French version of the SF-36; bivariate analyses and exploratory multivariate linear regressions were performed.

**Results:**

The mean age was 54.4 ± 8.9 years; cervical cancer accounted for 85.6% of cases, and 82.2% of patients had advanced disease. HRQoL showed a dissociated profile, with severe physical impairment (mean PCS 33.3 ± 3.2) and a mental component within the normative range (mean MCS 49.4 ± 4.5). In multivariate exploratory analysis, lower PCS was associated with advanced age (*p* < 0.001), advanced stage (*p* = 0.014), and ECOG ≥ 2 (*p* = 0.005). In the MCS model, advanced stage (*p* < 0.001) and severe pain (*p* = 0.004) showed positive associations, a counterintuitive finding that should be interpreted cautiously.

**Conclusion:**

In our sample, Djiboutian women with gynecological cancers experienced substantial physical impairment in HRQoL, while the mental component appeared relatively preserved. These results underscore the need for earlier diagnosis, better pain management, supportive care, and systematic integration of HRQoL into oncological care.

**Supplementary Information:**

The online version contains supplementary material available at https://doi.org/10.1186/s12905-026-04755-x.

## Introduction

Gynecological cancers including cervical, endometrial, and ovarian cancers remain a major public health issue due to their prevalence, severity, and deep impact on women’s health and daily life [1–3]. In 2022, the global cancer burden approached 20 million new cases and 9.7 million deaths, with a disproportionate share of preventable mortality occurring in low- and middle-income countries [1, 2]. Cervical cancer particularly exemplifies these disparities: it is largely preventable through human papillomavirus vaccination, screening, and early treatment of precancerous and invasive lesions, yet its burden remains high where these interventions are least accessible [3–6]. In sub-Saharan Africa, late presentation and disruptions in the continuum of care are closely linked to low screening coverage, delays in diagnostic confirmation, limited access to pathology and imaging, as well as inadequate availability of essential treatments, including radiotherapy, brachytherapy, and palliative care [6–13]. In Djibouti, available estimates mainly rely on international models rather than a national population-based cancer registry, which limits local data useful for care planning [7, 8]. In this context, studies focused on patient experience are especially important, as they document aspects often missing from routine clinical indicators. Indeed, health-related quality of life (HRQoL) is now recognized as an essential outcome criterion in oncology because it allows for assessing the impact of the disease, symptoms, and treatments on patients’ physical, psychological, social, and functional well-being [14–22]. Gynecological cancers may also affect body image, sexuality, marital relationships, family roles, social participation, and financial security, particularly in settings where supportive care remains insufficiently structured [14, 15, 20–24]. Among the available instruments, the Short Form-36 Health Survey (SF-36) is one of the most widely used generic tools for measuring HRQoL; it covers eight domains and enables calculation of two standardized summary scores: the Physical Component Summary (PCS) and the Mental Component Summary (MCS) [17–19]. Its validated French version permits use in Francophone settings, though the lack of local norms is a significant limitation for interpretation [19]. A generic instrument was preferred over cancer-specific tools such as the EORTC QLQ-C30 and its gynecological modules (QLQ-CX24, QLQ-OV28, QLQ-EN24), as an accessible, cost-free validated version of these disease-specific instruments could not be secured in this setting at the time of the study; this choice is revisited as a limitation in the Discussion. In this context, the present study aimed to assess HRQoL among Djiboutian women with gynecologic cancers using the validated French version of the SF-36, to describe standardized PCS and MCS scores, and to examine their associations with selected sociodemographic and clinical characteristics. By identifying factors associated with physical and mental HRQoL, this study seeks to inform patient-centered oncologic care, supportive interventions, and future research in the Djiboutian context and in comparable resource-limited settings.

## Methodology

### Study design and setting

This was an analytical cross-sectional study with prospective data collection, conducted in Djibouti from February to April 30, 2024. Recruitment took place in two referral facilities involved in the management and referral of gynecological pathologies: the National Police Hospital and the Housseina Reproductive Health Center. These two sites were selected for their role in identifying, monitoring, and referring women with confirmed gynecological cancers.

### Study population, eligibility criteria, and sampling

**Fig. 1:**
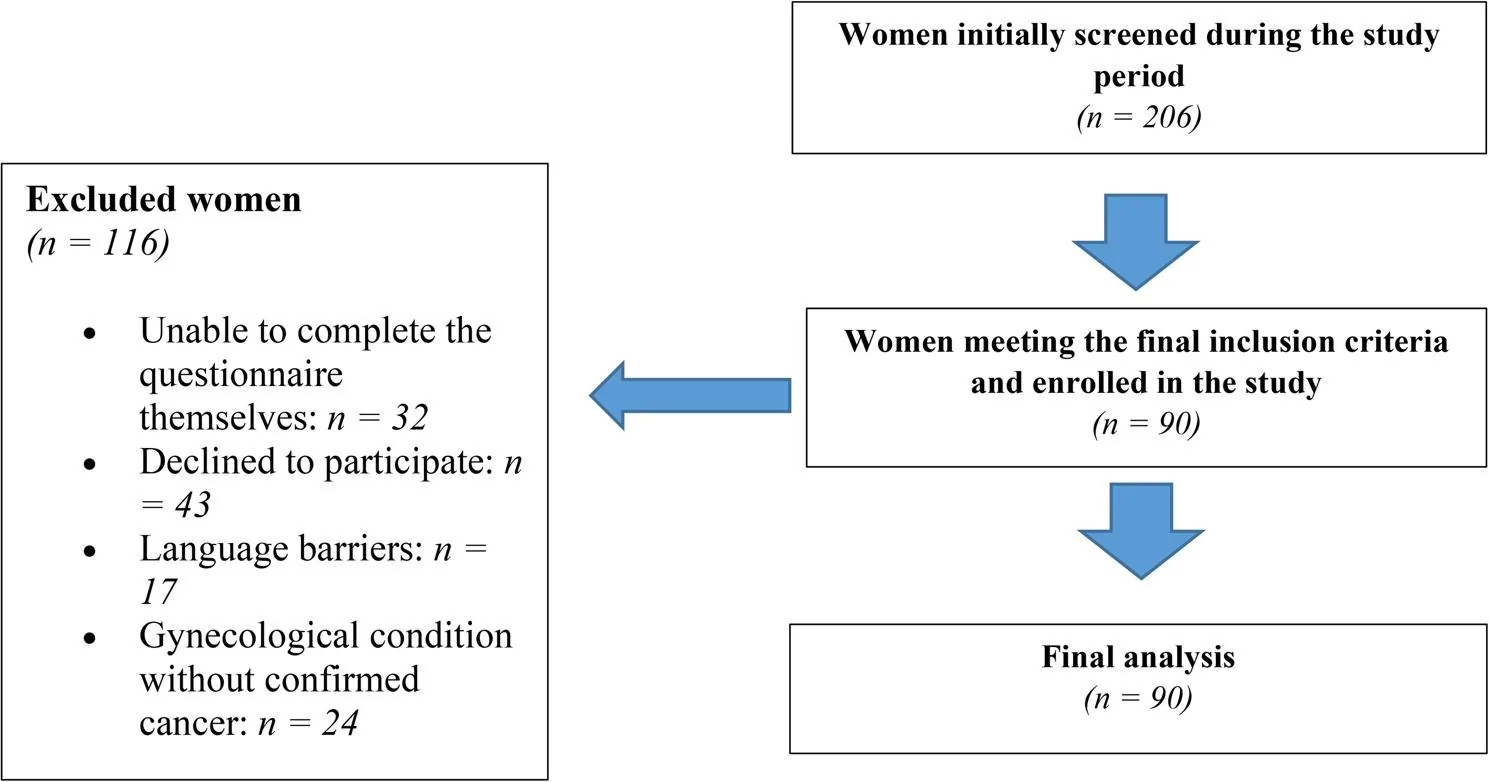
Flow chart of participant selection

The source population consisted of Djiboutian women followed at the two reference centers for a gynecological cancer of the reproductive tract, mainly cervical, endometrial, and ovarian cancers. Eligible participants were women aged 35 to 75 years (an age range selected to reflect the typical age distribution of cervical, endometrial, and ovarian cancer diagnoses reported in the epidemiological literature), with a diagnosis of gynecological cancer confirmed by histological examination, capable of responding to study questions (assessed through the same clinical evaluation used to rule out the major neurological or psychiatric disorders listed among the exclusion criteria below, rather than as a separate, informal judgement), and having given informed consent. Women with major neurological or psychiatric disorders likely to interfere with participation, a language barrier preventing adequate communication, or who refused to participate were excluded. Sampling was non-probabilistic and consecutive. Among the 206 women assessed during the study period, 116 were excluded: 32 were unable to respond to the questionnaire, 43 refused to participate, 17 had a language barrier, and 24 had a gynecological condition without histologically confirmed cancer. The final analysis therefore included 90 women. No formal sample size calculation was performed prior to the study, as the objective was exploratory and descriptive-analytical; the final sample corresponded to all eligible women recruited during the data collection period. The participant selection process is shown in Fig. 1.

### Data collection

Data were collected through individual face-to-face interviews conducted by the principal investigator. All interviews were conducted in a private setting (a dedicated office), without a third party present, and were administered by the same single investigator throughout the study, limiting between-interviewer variability in questionnaire administration. Health-related quality of life was assessed using the validated French version of the SF-36 questionnaire. Sociodemographic, clinical and care pathway variables not covered by the SF-36 were collected using a pre-tested structured data collection form developed specifically for this study. When necessary, questions were reformulated in the local language to ensure proper understanding. This reformulation was performed ad hoc by the interviewer, based on her judgement of each participant’s understanding, rather than following a pre-established, standardized translation or cultural adaptation protocol; this represents a limitation of the study, discussed further below. The variables collected included age, marital status, education level, occupation, cancer type, FIGO stage, treatments received, comorbidities, diagnostic delay, time since diagnosis, distance between home and the referral center, family support, pain intensity, and ECOG performance status.

### Assessment of HRQoL, SF-36 scoring and study variables

HRQoL was assessed using the validated French version of the 36-Item Short Form Health Survey (SF-36) questionnaire [19]. The SF-36 was not developed specifically for this study; it is a previously published and widely used generic instrument for assessing health-related quality of life [17, 18]. Scores for the eight domains were calculated according to the standard scoring procedures described by Ware and colleagues [17, 18]. Each domain ranges from 0 to 100, with higher scores indicating better quality of life. The summary scores PCS and MCS were calculated using the norm-based scoring method, including z standardization, weighted aggregation, and transformation into T scores with a reference mean of 50 and standard deviation of 10 [18]. Scores below 50 were interpreted as indicating an HRQoL below the reference norm. Sociodemographic, clinical and care pathway variables not covered by the SF-36 were collected using a structured data collection form developed specifically for this study. This form included information on age, marital status, education level, occupation, cancer type, FIGO stage, treatment received, comorbidities, diagnostic delay, time since diagnosis, distance from home to the healthcare facility, family support, ECOG performance status, and pain intensity. The English version of this structured data collection form is provided as Supplementary File 1. This form was piloted with a small number of patients prior to the start of data collection to check the clarity and relevance of the items; no changes to the form were required. The main dependent variables were the PCS and MCS scores. The secondary dependent variables were the scores for the eight SF-36 domains. The explanatory variables included age, marital status, education level, occupation, cancer type, FIGO stage, categorized as early stage (I-II) or advanced stage (III-IV) according to gynecological oncology staging frameworks and guidelines [8, 25–27], treatments received, comorbidities, diagnostic delay, time since diagnosis, distance to the referral center, family support, ECOG performance status, and pain intensity. Pain was assessed using a visual analogue scale from 0 to 10 and classified as moderate pain (4–6) or severe pain (7–10). Functional status was assessed using the ECOG scale [28].

### Statistical analysis

Qualitative variables were summarized using counts and percentages. Quantitative variables were described by means with standard deviations and by medians where appropriate. The normality of continuous variables was assessed using the Shapiro-Wilk test. Comparisons between two groups were performed with the Mann-Whitney U test, while comparisons between more than two groups were carried out with the Kruskal-Wallis test. Associations between continuous variables were assessed by Spearman’s rank correlation coefficient. Missing data mainly concerned treatment-related variables, with 29 to 32 missing observations depending on the variable. No imputation was performed; analyses involving these variables were therefore conducted on complete cases and interpreted with caution. A post hoc comparison of patients with and without missing treatment data found no significant association between missingness and disease stage, ECOG status, pain intensity, or age (all *p* > 0.20), suggesting that missingness was not related to these major clinical severity markers; however, patients with missing treatment data had a slightly higher PCS than complete cases (34.2 ± 3.6 vs. 32.8 ± 2.9; *p* = 0.024), with no corresponding difference in MCS (*p* = 0.238). This indicates that missingness was not entirely unrelated to physical HRQoL, and complete-case treatment analyses should be interpreted with this in mind. Associations between selected variables and PCS/MCS scores were explored, on an exploratory basis, using separate multivariate linear regression models. Variables were included in the models if they were clinically relevant and/or associated with the outcome with *p* < 0.20 in bivariate analysis. Adjusted beta coefficients, 95% confidence intervals, and p values were reported. No a priori sample size or power calculation was performed for these regression models, and some reference categories included as few as 5–6 patients; the multivariable analyses should therefore be considered exploratory, and associations are reported as such rather than as independent determinants (see Limitations). Diagnostic checks were performed for both regression models, including variance inflation factors (VIF) to assess multicollinearity among predictors, the Shapiro-Wilk test and visual inspection for residual normality, the Breusch-Pagan test for homoscedasticity, and Cook’s distance to identify influential observations (see Results and Table 4 note). The threshold for statistical significance was set at *p* < 0.05. Analyses were performed with Python version 3.12, using SciPy and Statsmodels. The reporting of this observational study was guided by the STROBE recommendations [29].

### Ethical considerations

This study was conducted in accordance with the ethical principles governing research involving human participants, including the Declaration of Helsinki, the CIOMS international ethical guidelines, and WHO recommendations. Administrative authorizations permitting access to participants and clinical data were obtained from both participating health facilities in Djibouti prior to the start of the study; these facility-level authorizations were administrative in nature and did not constitute ethical review. In parallel, a request for ethical review was submitted to the National Ethics Committee for Medical Research of the National Institute of Public Health of Djibouti (INSPD), Ministry of Health, the competent national authority for the ethical review of medical research in Djibouti, before data collection began. The Committee’s evaluation of the protocol was completed after data collection had concluded, issuing a favourable ethical opinion under reference No. 145/INSPD/2026 on 10 June 2026. The authors acknowledge that proceeding with data collection before this formal approval was obtained constitutes a protocol deviation. The study was observational and non-interventional throughout, involving no experimental procedure, biological sampling, or modification of participants’ clinical care; it relied on a validated, non-invasive questionnaire administered with each participant’s oral informed consent, and all data were anonymised.

Before inclusion, all participants received clear oral information about the objectives and procedures of the study, the voluntary nature of participation, the confidentiality and anonymization of their data, and their right to decline participation or withdraw at any time without any consequence for their medical care. Given that a substantial proportion of participants had no formal schooling and limited literacy, oral informed consent was obtained from all participants before questionnaire administration. Consent was obtained in a private, one-on-one setting between the participant and the principal investigator, without a third-party witness; each participant’s consent was documented by a dedicated checkbox on the structured data collection form, completed by the investigator at the time of interview. The requirement for informed consent was not waived. All responses were anonymised, and confidentiality was strictly maintained throughout the study.

## Results

### Sociodemographic and clinical characteristics

A total of 90 patients were included. The mean age was 54.4 ± 8.9 years, with a range from 35 to 75 years. Cervical cancer was the predominant diagnosis, accounting for 85.6% of cases, followed by endometrial cancer and ovarian cancer. Overall, 82.2% of patients had advanced disease (FIGO stages III-IV), among whom 73.3% were classified as stage III. Regarding diagnostic intervals, 52.2% of participants reported an interval of approximately one year between the onset of symptoms and confirmation of the diagnosis, while 23.3% reported a delay of at least two years. The time elapsed since diagnosis was about one year for 40.0% of participants and at least two years for 34.4%. Socioeconomically, 88.9% of women had not received formal education and 93.3% were unemployed. Family support was reported by 84.4% of participants. Clinically, 93.3% had an ECOG score ≥ 2 and 94.4% reported severe pain on the visual analogue scale. These characteristics are summarized in Table 1.

**Table 1:** Sociodemographic and clinical characteristics of the study participants

Variable	*n* (%)
Demographic characteristics	
Total sample	90 (100)
Mean age, years ± SD [range]	54.4 ± 8.9 [35–75]
Cancer type	
Cervical cancer	77 (85.6)
Endometrial cancer	8 (8.9)
Ovarian cancer	5 (5.6)
FIGO stage	
Stage I	4 (4.4)
Stage II	12 (13.3)
Stage III	66 (73.3)
Stage IV	8 (8.9)
Early-stage disease (I–II)	16 (17.8)
Advanced-stage disease (III–IV)	74 (82.2)
Treatment received	
Chemotherapy	50 (55.6)*
Surgery	12 (13.3)*
Radiotherapy	0 (0)
Hormone therapy	0 (0)
Comorbidities	
At least one comorbidity	28 (31.1)
No comorbidity	62 (68.9)
Time since diagnosis	
6 months	23 (25.6)
1 year	36 (40.0)
2 years	19 (21.1)
3 years	12 (13.3)
Diagnostic delay	
≤ 6 months	22 (24.4)
1 year	47 (52.2)
≥ 2 years	21 (23.3)
Distance from home to healthcare facility	
≤ 10 km	72 (80.0)
11–100 km	8 (8.9)
> 100 km	10 (11.1)
ECOG performance status	
ECOG 0–1	6 (6.7)
ECOG ≥ 2	84 (93.3)
Pain intensity, VAS	
Moderate pain, VAS 4–6	5 (5.6)
Severe pain, VAS 7–10	85 (94.4)
Socioeconomic characteristics	
Married	69 (76.7)
Widowed	16 (17.8)
Divorced	5 (5.6)
No formal education	80 (88.9)
Formal education	10 (11.1)
Unemployed	84 (93.3)
Employed	6 (6.7)
Family support: yes	76 (84.4)

*ECOG* Eastern Cooperative Oncology Group, *VAS* visual analog scale, *SD* standard deviation

*Incomplete treatment data, not reported for 29 to 32 patients

### SF-36 domain scores and PCS/MCS summary scores

The SF-36 results revealed a dissociated HRQoL profile, characterized by severe impairment of the physical component and relative preservation of the mental component. Among the physical domains, the lowest scores were observed for physical functioning (12.1 ± 9.9) and bodily pain (10.0 ± 11.3). General health was less impaired, while the high physical role score likely reflected a ceiling effect rather than truly preserved physical functioning. This is consistent with the coarse, dichotomous (yes/no) item format used to score the Role-Physical domain, which is well documented to produce skewed distributions with substantial floor and ceiling effects across diverse populations, largely independent of true underlying functional status [17, 18]; in addition, because 93.3% of participants were unemployed, items asking about limitations relative to a person’s usual work or activities may have had limited applicability in this population, further contributing to the apparent ceiling. Among the mental domains, mental health was impaired and vitality moderately reduced, whereas social functioning appeared relatively preserved. The mean PCS score was 33.3 ± 3.2, that is, 1.7 standard deviations below the normative mean, while the mean MCS score was 49.4 ± 4.5 and remained within the normative range. These scores are presented in Table 2.

**Table 2:** SF-36 domain scores and physical and mental component summary scores (PCS and MCS) (*N* = 90)

SF-36 domain	Mean ± SD	Median	Min-max	Interpretation
Physical component				
Physical functioning (PF)	12.1 ± 9.9	10.0	0.0–35.0	Severely impaired
Role limitations due to physical health (RP)	97.5 ± 7.5	100.0	75.0–100.0	Preserved (ceiling effect)
Bodily pain (BP)	10.0 ± 11.3	0.0	0.0–33.3	Severely impaired
General health (GH)	75.8 ± 4.5	75.0	65.0–80.0	Relatively preserved
Mental component				
Vitality (VT)	48.3 ± 4.0	50.0	40.0–55.0	Moderately impaired
Social functioning (SF)	77.8 ± 7.9	75.0	75.0–100.0	Relatively preserved
Role limitations due to emotional problems (RE)	86.3 ± 27.8	100.0	0.0–100.0	Heterogeneous
Mental health (MH)	43.1 ± 5.8	44.0	32.0–60.0	Impaired
Standardized summary scores				
Physical component summary (PCS)	33.3 ± 3.2	32.5	28.3–42.6	Severely impaired
Mental component summary (MCS)	49.4 ± 4.5	51.1	34.0–55.8	Within the normative range

Standardized T scores: reference norm = 50 ± 10; a score < 50 indicates an HRQoL below the reference norm

### Factors associated with PCS and MCS scores in bivariate analysis

In bivariate analysis, advanced stage was associated with a lower PCS (32.4 ± 2.5 vs. 37.4 ± 2.9; *p* < 0.001), but with a higher MCS (50.7 ± 3.1 vs. 43.2 ± 5.0; *p* < 0.001), compared to early stage. A similar pattern was observed for ECOG index: women with ECOG ≥ 2 had a lower PCS (32.8 ± 2.7 vs. 39.8 ± 1.7; *p* < 0.001) and a higher MCS (50.0 ± 3.9 vs. 41.1 ± 4.2; *p* < 0.001). Severe pain was associated with a lower PCS (33.0 ± 2.9 vs. 39.0 ± 2.6; *p* = 0.001), consistent with its expected impact on physical health. However, as with advanced stage and ECOG ≥ 2, severe pain was associated with a higher MCS (50.0 ± 3.8 vs. 39.3 ± 3.5; *p* < 0.001), extending the same paradoxical pattern to a third clinical severity indicator. Women with formal education had a higher PCS than those without formal schooling (36.8 ± 3.1 vs. 32.9 ± 3.0; *p* = 0.001), with no significant association with MCS (*p* = 0.055). Family support was not significantly associated with either PCS or MCS. In complete-case analyses, chemotherapy was associated with a lower PCS (*p* = 0.009), while surgery was associated with a higher PCS (*p* = 0.010); these treatment-related findings should be interpreted with caution due to missing data, as patients with missing treatment data had slightly higher PCS scores than complete cases (see Methods). The bivariate comparisons are summarized in Table 3.

**Table 3:** Comparison of PCS and MCS scores according to selected clinical and sociodemographic factors

Variable	PCS group 1 (1st-named group)	PCS group 2 (2nd-named group)	*p*-value	MCS group 1 (1st-named group)	MCS group 2 (2nd-named group)	*p*-value
FIGO stage: early-stage disease I–II vs. advanced-stage disease III–IV	37.4 ± 2.9	32.4 ± 2.5	< 0.001	43.2 ± 5.0	50.7 ± 3.1	< 0.001
ECOG performance status: 0–1 vs. ≥ 2	39.8 ± 1.7	32.8 ± 2.7	< 0.001	41.1 ± 4.2	50.0 ± 3.9	< 0.001
Pain intensity: moderate vs. severe	39.0 ± 2.6	33.0 ± 2.9	0.001	39.3 ± 3.5	50.0 ± 3.8	< 0.001
Educational level: formal education vs. no formal education	36.8 ± 3.1	32.9 ± 3.0	0.001	45.4 ± 6.4	49.9 ± 4.0	0.055
Family support: yes vs. no	33.5 ± 3.2	32.1 ± 2.8	0.094	49.2 ± 4.7	50.8 ± 3.2	0.273
Chemotherapy: yes vs. no, complete cases	32.3 ± 2.9	34.9 ± 2.6	0.009	50.3 ± 4.1	48.2 ± 3.9	0.073
Surgery: yes vs. no, complete cases	34.5 ± 2.8	32.1 ± 2.9	0.010	48.6 ± 4.0	50.6 ± 3.9	0.089

Treatment-related variables were analysed using complete cases only. For each row, the 1st-named group corresponds to the first category listed in the Variable column (e.g., early-stage disease, ECOG 0–1, moderate pain) and the 2nd-named group to the second category (e.g., advanced-stage disease, ECOG ≥ 2, severe pain)

*PCS* Physical Component Summary, *MCS* Mental Component Summary, *ECOG* Eastern Cooperative Oncology Group

### Comparison of SF-36 domains according to tumor stage

Patients with advanced disease had lower physical functioning scores than those with early-stage disease (10.3 vs. 20.3; *p* < 0.001) and markedly lower bodily pain scores (6.6 vs. 25.7; *p* < 0.001). Conversely, advanced disease was associated with higher, not lower, scores for vitality (49.3 vs. 43.8; *p* < 0.001) and emotional role (94.6 vs. 47.9; *p* < 0.001), consistent with the same paradoxical pattern discussed for the MCS score (The MCS paradox: progressive psychological adaptation and response shift section). In contrast, no statistically significant differences were observed for general health (*p* = 0.090), social functioning (*p* = 0.123), or mental health (*p* = 0.242).

### Spearman correlations

Age was negatively correlated with PCS (*r* = -0.569; *p* < 0.001) and positively correlated with MCS (r = + 0.448; *p* < 0.001). Similarly, time since diagnosis was negatively correlated with PCS (*r* = -0.473; *p* < 0.001) and positively correlated with MCS (r = + 0.397; *p* < 0.001). Diagnostic delay followed the same pattern, with a negative correlation with PCS (*r* = -0.474; *p* < 0.001) and a positive correlation with MCS (r = + 0.367; *p* < 0.001).

### Exploratory multivariate analysis

In the multivariate model for PCS (adjusted R² = 0.501), advanced age (β = -0.13; *p* < 0.001), advanced stage (β = -2.22; *p* = 0.014), and ECOG ≥ 2 (β = -3.41; *p* = 0.005) were associated with lower PCS scores. In the multivariate model for MCS (adjusted R² = 0.500), advanced age (β = +0.12; *p* = 0.016), advanced stage (β = +4.61; *p* < 0.001), and severe pain (β = +5.23; *p* = 0.004) were associated with higher MCS scores. The positive coefficients observed for advanced stage and severe pain may reflect a process of progressive psychological adaptation, as discussed below. The results of the models are presented in Table 4.

**Table 4:** Factors associated with PCS and MCS scores in multiple linear regression analysis

Variable	Adjusted β	95% CI	*p*-value
PCS model: *R*² = 0.535; adjusted *R*² = 0.501			
Age, years	−0.13	[− 0.20; −0.06]	< 0.001
Advanced-stage disease, FIGO III–IV	−2.22	[− 3.99; −0.46]	0.014
ECOG ≥ 2	−3.41	[− 5.78; −1.05]	0.005
Severe pain, VAS 7–10	−1.54	[− 4.02; +0.94]	0.220
No formal education	+ 0.28	[− 1.55; +2.11]	0.763
Family support, yes	−0.28	[− 1.77; +1.20]	0.704
MCS model: R² = 0.534; adjusted R² = 0.500			
Age, years	+ 0.12	[+ 0.02; +0.21]	0.016
Advanced-stage disease, FIGO III-IV	+ 4.61	[+ 2.12; +7.09]	< 0.001
ECOG ≥ 2	+ 2.79	[− 0.54; +6.12]	0.099
Severe pain, VAS 7–10	+ 5.23	[+ 1.75; +8.72]	0.004
No formal education	−1.66	[− 4.24; +0.92]	0.203
Family support, yes	+ 0.35	[− 1.74; +2.44]	0.739

*Note*: Adjusted β regression coefficient; *CI* confidence interval; *PCS* Physical Component Summary; *MCS* Mental Component Summary. Diagnostic checks: variance inflation factors ranged from 1.29 to 2.02 across predictors in both models, indicating no problematic multicollinearity. For the PCS model, residuals were approximately normally distributed (Shapiro-Wilk *p* = 0.219) and homoscedastic (Breusch-Pagan *p* = 0.866); 7 of 90 observations exceeded the Cook's distance threshold of 4/n, with a maximum value of 0.17. For the MCS model, residuals deviated from normality (Shapiro-Wilk *p* = 0.001) and showed evidence of heteroscedasticity (Breusch-Pagan *p* = 0.002); 11 of 90 observations exceeded the Cook's distance threshold, with a maximum value of 0.38. These diagnostics indicate that the MCS model is statistically less stable than the PCS model, reinforcing the exploratory, hypothesis-generating interpretation of the MCS associations recommended elsewhere in this manuscript

## Discussion

### A dissociated HRQoL profile : severe physical impairment and relative preservation of the mental component

The main finding of this study is the clear dissociation between the two composite scores of the SF-36. The mean PCS of 33.3, which is 1.7 standard deviations below the normative value, indicates severe physical impairment. In contrast, the mean MCS of 49.4 remains close to the reference norm. This profile differs from many cohorts in high-income countries, where physical and mental components are often impaired in parallel, but aligns with observations reported in several studies on gynecological cancers and cancer survivors in Africa, where the physical burden is often very significant while the mental component may be partially preserved [14–17, 20–22, 30, 31]. Similar patterns have been reported for other gynecologic cancer subtypes: a cross-sectional study of endometrioid endometrial cancer survivors found that physical functioning was reduced in association with comorbid factors such as obesity, while a high prevalence of sexual dysfunction persisted across the cohort, illustrating how the physical dimension of HRQoL can remain compromised across different gynecologic cancer types and survivorship contexts [32]. The severity of physical impairment observed in our cohort may be related to the convergence of several unfavorable factors: a high proportion of advanced stages, a very high prevalence of severe pain, and marked impairment of functional status. This convergence of clinical severity indicators is consistent with the broader gynecologic oncology literature on prognostic assessment, where markers of systemic inflammation, nutritional status, and perioperative risk are similarly associated with survival and functional outcomes [33, 34], underscoring the close relationship between clinical severity, functional status, and quality of life in this patient population. These results are consistent with African data showing the frequency of late diagnoses, the high proportion of advanced stages, and the impact of treatment delays on clinical outcomes [9–13, 16]. They are also in line with recent studies on the HRQoL of women with gynecological cancers, which identify pain, fatigue, physical limitations, and clinical severity as major determinants of quality of life [14, 15, 20–22, 30, 31].

### The MCS paradox: progressive psychological adaptation and response shift

The association between advanced stage, ECOG ≥ 2, and severe pain and higher MCS scores constitutes an apparently counterintuitive finding. One possible explanation is the existence of mechanisms for progressive psychological adaptation. Patients living with the disease for a longer period may develop cognitive, emotional, familial, or spiritual strategies that allow them to maintain a certain balance despite the progression of the disease. Recent studies on gynecologic cancers highlight the importance of social support, conjugal support, and psychological adjustment in the mental quality of life of patients [15, 23, 35, 36]. A second explanation is based on the concept of “response shift,” which refers to a modification of the internal standards, values, or expectations that patients use to assess their own quality of life. Within this framework, a patient facing advanced disease may report a relatively preserved mental component not because her objective situation is better, but because her subjective frame of evaluation has changed over the course of the illness [37]. However, this interpretation must remain cautious, as the cross-sectional study design does not allow direct observation of the temporal evolution of this adaptation. A third, more parsimonious explanation should also be considered: the standard norm-based scoring method used to derive PCS and MCS relies on an orthogonal factor-analytic model that forces the two summary scores to be statistically uncorrelated; this scoring method is documented to artificially inflate the MCS in patients with substantial physical disability, independently of any genuine psychological adaptation [38]. This statistical property of the scoring algorithm may therefore account, at least in part, for the paradoxical association between greater clinical severity and higher MCS observed here, and should temper a purely psychological interpretation based on response shift alone.

### Pain as a major factor associated with quality of life

Severe pain emerged as one of the factors most strongly associated with physical HRQoL. In bivariate and multivariate analyses, severe pain was strongly associated with poorer PCS scores, confirming its central role in patients’ physical experience of the disease. As with advanced stage and ECOG ≥ 2 (The MCS paradox: progressive psychological adaptation and response shift section), severe pain was paradoxically associated with a higher, not lower, MCS score; this unexpected direction should be interpreted with the same caution as the broader MCS paradox and should not be overinterpreted as evidence that pain improves mental well-being. This observation is consistent with recent data showing that pain, fatigue, and symptom burden are strongly associated with poorer quality of life in women with gynecologic cancers [14, 20–22, 30, 31]. In our cohort, 94.4% of women reported severe pain, suggesting a pressing need to strengthen pain assessment and management. International guidelines and palliative care data emphasize that pain relief and supportive care are essential components of oncologic care, particularly in contexts where patients are frequently diagnosed at an advanced stage [39]. In Djibouti, these results support a systematic approach to pain assessment, improved access to essential analgesics, and early integration of palliative and supportive care.12

### Age, tumor stage and functional status as factors associated with PCS

In multivariate analysis, advanced age, advanced stage and ECOG ≥ 2 remained associated with a decrease in PCS. These results are both biologically and clinically consistent: age can exacerbate functional vulnerability, advanced stage increases symptom burden, and ECOG directly reflects the patient’s ability to carry out daily activities [20, 21, 28]. The positive association observed between age and MCS could, for its part, reflect a better psychological coping ability in certain older patients, but this hypothesis requires longitudinal and qualitative studies to be confirmed.

### Level of education and family support: social determinants to be interpreted with caution

Level of education was associated with PCS in bivariate analysis, but this association disappeared after adjustment. This suggests that its apparent effect could be mediated by other clinical or social variables, such as age, tumor stage or functional status. Nevertheless, the very high proportion of women without formal education underscores the importance of therapeutic education strategies adapted to health literacy levels and sociocultural context. Recent African studies show that socioeconomic factors, financial toxicity, and access to care have a strong impact on the experiences of patients with gynecological cancers [14, 16, 20]. Family support, although reported by the majority of participants, was not significantly associated with PCS or MCS. This lack of association must be interpreted with caution, since the measure used was binary and did not allow assessment of the quality, intensity, or concrete forms of support. Yet recent literature indicates that spousal, social, and family support may play an important protective role in the quality of life and emotional adjustment of women with gynecological cancers [15, 23, 35]. Future studies should use more refined instruments to explore this dimension.

### Study limitations

Several limitations must be acknowledged. First, the cross-sectional design of the study does not allow any causal relationship to be established between the variables studied and the HRQoL scores. Moreover, the non-probabilistic, consecutive sampling from only two referral centres, together with a substantial refusal rate (43 of the 206 women assessed), limits the generalizability of the findings and may have introduced selection bias. The direction of this bias cannot be determined with certainty, but it is plausible that women with poorer health status, more severe disease, lower literacy, or worse quality of life were less likely to complete the questionnaire (32 of the 116 excluded women were unable to respond, and a further 17 had a language barrier); if so, the HRQoL burden reported here may under-estimate that of the broader source population of women with gynecological cancer in these two centres. No sociodemographic or clinical data were retained for women who were screened but not included, precluding a formal comparison of included and excluded women; this is acknowledged as a data availability limitation. In addition, cervical cancer accounted for 85.6% of the cohort, with endometrial (8.9%) and ovarian (5.6%) cancers markedly under-represented; the findings therefore primarily reflect the HRQoL experience of women with cervical cancer, and should not be assumed to generalize to women with endometrial or ovarian cancer specifically. Second, although all eligible patients recruited during the study period were included, the sample size remains limited, with small numbers in certain subgroups, particularly in patients with ECOG 0–1. Several reference categories used in the regression models included as few as 5–6 patients, and no formal sample size calculation was performed prior to the study, since the objective was exploratory and descriptive-analytical. Regression coefficients based on such small and imbalanced subgroups are inherently unstable, and the multivariable models presented here should be regarded as exploratory, hypothesis-generating analyses rather than confirmatory tests; the term “associated factors” has therefore been used throughout in preference to “independent determinants”, and formal sample-size and power considerations should inform any confirmatory follow-up study. Consistent with this exploratory framing, diagnostic checks (Table 4 note) showed that the MCS model exhibited non-normal residuals and heteroscedasticity, unlike the PCS model; the MCS associations, including the pain and stage paradox discussed above, should accordingly be interpreted with particular caution. Third, the clinical homogeneity of the cohort, characterized by a high proportion of advanced stages and severe pain, may have reduced variability and limited the detection of finer associations. Other methodological limitations must be considered. As a generic instrument, the SF-36 does not capture domains of particular relevance to gynecological cancer, such as sexuality, body image, treatment-related toxicity, lymphedema, vaginal symptoms, or financial toxicity; disease-specific instruments such as the EORTC QLQ-C30 with its gynecological modules (QLQ-CX24, QLQ-OV28, QLQ-EN24) would likely have captured these dimensions more fully, but an accessible, cost-free validated version of these instruments could not be secured in this setting. Oral administration of the SF-36, necessary due to the low educational level of many participants, may have introduced interviewer-related bias. In particular, the ad hoc reformulation of questions in the local language, performed by the interviewer without a pre-established, standardized translation or cultural adaptation protocol, may have introduced inconsistencies in how items were understood across participants and cannot be assumed to be measurement-equivalent to the validated French version; this is an important limitation for the interpretation of individual domain scores. Future studies in this setting would benefit from a formally translated and culturally adapted local-language version of a disease-specific instrument, developed and validated prior to data collection. The absence of local norms for PCS and MCS scores required the use of U.S. reference norms, which may limit the cultural comparability of the results [18, 19]. Missing data, notably for variables related to treatment, reduced the interpretability of certain analyses. Furthermore, multiple bivariate comparisons were performed across the eight SF-36 domains, the PCS/MCS summary scores, and numerous clinical and sociodemographic variables, without formal adjustment for multiple testing; some statistically significant associations may therefore reflect chance findings, and results should be interpreted as hypothesis-generating. Finally, the possible existence of a response shift phenomenon complicates the interpretation of the mental component among patients with advanced disease [37]. Importantly, although a request for ethical review was submitted to the National Ethics Committee before data collection began, the Committee’s formal approval was not issued until more than two years later, after data collection had already taken place; data collection therefore proceeded before formal approval was obtained. This delay reflects the Committee’s own processing time during a period of progressive institutional structuring, rather than a failure by the study team to seek prospective review, but it nonetheless constitutes a protocol deviation, which we report transparently for the reader’s and Editor’s judgment. Despite these limitations, the study was reported in accordance with STROBE recommendations [29].

### Perspectives

These results support several priorities for practice and research. In clinical and public health terms, they highlight the need to strengthen early diagnosis, improve access to essential treatments, develop effective pain management, expand palliative and supportive care, and implement therapeutic education interventions tailored to the sociocultural characteristics of patients [6, 9–13, 20, 39]. The systematic integration of HRQoL assessment into oncology care could help better identify unmet needs, guide clinical decision making, and monitor patient-centered outcomes [14, 17–19]. In terms of research, longitudinal studies are needed to understand the evolution of HRQoL throughout the care pathway, clarify temporal relationships between disease, treatment, pain, and psychological adaptation, and better interpret the dissociated profile observed in this study. Larger-scale multicenter studies would also improve statistical power, explore differences by cancer type, and develop local reference norms for interpreting the SF-36 in Djibouti.

## Conclusion

In this cohort, Djiboutian women with gynecological cancers experienced significant impairment in health-related quality of life, characterized by severe deterioration of the physical component alongside relative preservation of the mental component. This dissociated profile is an important finding in the Djiboutian context. Advanced age, advanced disease stage, and impaired functional status were the main factors associated with lower physical HRQoL. Severe pain was highly prevalent and strongly associated with poorer physical HRQoL; however, its unexpected positive association with the mental component paralleling the pattern observed for advanced stage and ECOG ≥ 2 requires cautious interpretation and should not be overinterpreted; consistent with the exploratory nature of the multivariable analyses, this association should be regarded as hypothesis-generating only. These findings support the need for more integrated oncological care in resource-limited settings, combining earlier diagnosis, effective pain management, palliative and supportive care, psychosocial support, and regular HRQoL assessment.

## Supplementary Information

Supplementary Material 1.

Supplementary Material 2.

## Data Availability

No datasets were generated or analysed during the current study.

## Abbreviations

ECOG: Eastern Cooperative Oncology Group
FIGO: International Federation of Gynecology and Obstetrics
HRQoL: health-related quality of life
MCS: Mental Component Summary
PCS: Physical Component Summary
SF-36: 36-Item Short Form Health Survey
VAS: Visual analogue scale
