# Supplementary material for: Health-related quality of life among Djiboutian women with gynecological cancers, predominantly cervical cancer : a cross-sectional analytical study
Source: BMC Womens Health. 2026 Jul 31;26:561. doi: 10.1186/s12905-026-04755-x (PMC13711914; doi:10.1186/s12905-026-04755-x)
Supplement: Supplementary file 1 [file 12905_2026_4755_MOESM1_ESM.docx]

**Supplementary File 1**

**Structured Sociodemographic, Clinical and Care Pathway Data Collection Form**

*Study: Health-related quality of life among Djiboutian women with gynecological cancers: an analytical cross-sectional study*

Note: This structured form was developed specifically for the present study to record sociodemographic, clinical and care pathway variables not covered by the SF-36 questionnaire. Health-related quality of life was assessed separately using the published French version of the SF-36.

# Administrative information

| **Participant study code** | __________ |
| --- | --- |
| **Data collection date** | ____ / ____ / ______ |
| **Recruitment site / department** | ____________________________ |
| **Interviewer code** | __________ |
| **Data source** | ☐ Participant interview ☐ Medical record ☐ Both |

# A. Sociodemographic characteristics

| **Variable** | **Response / coding** | **Notes** |
| --- | --- | --- |
| **Age** | ____ years | Record exact age if available. |
| **Age group** | ☐ <40 ☐ 40–49 ☐ 50–59 ☐ ≥60 |  |
| **Place of residence** | ☐ Djibouti-ville ☐ Outside Djibouti-ville ☐ Other: ______ |  |
| **Marital status** | ☐ Single ☐ Married ☐ Divorced/separated ☐ Widowed |  |
| **Education level** | ☐ No formal education ☐ Primary ☐ Secondary ☐ Higher education |  |
| **Occupation** | ☐ Employed ☐ Unemployed ☐ Housewife ☐ Retired ☐ Other: ______ |  |
| **Health coverage / financial support** | ☐ Yes ☐ No ☐ Unknown | If applicable according to available data. |
| **Family/social support** | ☐ Yes ☐ No ☐ Limited ☐ Unknown |  |

# B. Clinical characteristics

| **Variable** | **Response / coding** | **Notes** |
| --- | --- | --- |
| **Type of gynecological cancer** | ☐ Cervical ☐ Ovarian ☐ Endometrial ☐ Vulvar ☐ Vaginal ☐ Other: ______ |  |
| **Histological confirmation** | ☐ Yes ☐ No ☐ Not available |  |
| **Histological type** | ____________________________ | If available. |
| **FIGO stage** | ☐ I ☐ II ☐ III ☐ IV ☐ Unknown/not documented |  |
| **Metastatic disease** | ☐ Yes ☐ No ☐ Unknown |  |
| **Date or period of diagnosis** | ____ / ____ / ______ or month/year: ______ |  |
| **Time since diagnosis** | ____ months |  |
| **Comorbidities** | ☐ None ☐ Hypertension ☐ Diabetes ☐ HIV ☐ Other: ______ |  |
| **ECOG performance status** | ☐ 0 ☐ 1 ☐ 2 ☐ 3 ☐ 4 ☐ Not assessed | Use available clinical assessment. |
| **Pain intensity** | VAS/NRS score: ____ /10 | 0=no pain; 1–3=mild; 4–6=moderate; 7–10=severe. |
| **Main symptoms reported** | ☐ Bleeding ☐ Pelvic pain ☐ Fatigue ☐ Urinary symptoms ☐ Digestive symptoms ☐ Other: ______ |  |

# C. Care pathway characteristics

| **Variable** | **Response / coding** | **Notes** |
| --- | --- | --- |
| **First symptoms** | ☐ Bleeding ☐ Pain ☐ Discharge ☐ Abdominal distension ☐ Other: ______ |  |
| **Date/period of first symptoms** | ____ / ____ / ______ or month/year: ______ | If exact date unavailable, record approximate month/year. |
| **Date/period of first consultation** | ____ / ____ / ______ or month/year: ______ |  |
| **Date/period of diagnostic confirmation** | ____ / ____ / ______ or month/year: ______ |  |
| **Date/period of treatment initiation** | ____ / ____ / ______ or month/year: ______ |  |
| **Patient interval** | ____ days/weeks/months | Time from first symptoms to first consultation. |
| **Diagnostic interval** | ____ days/weeks/months | Time from first consultation to diagnosis. |
| **Treatment interval** | ____ days/weeks/months | Time from diagnosis to treatment initiation. |
| **First point of care** | ☐ Primary health centre ☐ Hospital ☐ Private clinic ☐ Traditional/other ☐ Unknown |  |
| **Number of facilities consulted before diagnosis** | ____ facilities |  |
| **Referral to specialized care** | ☐ Yes ☐ No ☐ Unknown |  |
| **Reported barriers to care** | ☐ Financial ☐ Distance/transport ☐ Administrative ☐ Lack of information ☐ Waiting time ☐ Other: ______ | Multiple responses possible. |
| **Continuity of follow-up** | ☐ Regular ☐ Irregular ☐ Lost to follow-up risk ☐ Unknown |  |

# D. Treatment and supportive care

| **Variable** | **Response / coding** | **Notes** |
| --- | --- | --- |
| **Treatment received** | ☐ Surgery ☐ Chemotherapy ☐ Radiotherapy ☐ Hormonal therapy ☐ Palliative/supportive care ☐ None yet | Multiple responses possible. |
| **Treatment status** | ☐ Completed ☐ Ongoing ☐ Interrupted ☐ Not started ☐ Unknown |  |
| **Radiotherapy pathway** | ☐ Not indicated ☐ Local ☐ Referred abroad ☐ Not accessible ☐ Unknown | If relevant. |
| **Pain management** | ☐ Yes ☐ No ☐ Unknown | Analgesics or other pain-control measures. |
| **Psychosocial support** | ☐ Yes ☐ No ☐ Unknown |  |
| **Palliative care involvement** | ☐ Yes ☐ No ☐ Unknown |  |
| **Information received about disease/treatment** | ☐ Adequate ☐ Partial ☐ Insufficient ☐ Unknown | According to participant report. |
| **Family accompaniment during care** | ☐ Yes ☐ No ☐ Limited ☐ Unknown |  |

# E. Additional notes

______________________________________________________________________________________________

______________________________________________________________________________________________

______________________________________________________________________________________________

______________________________________________________________________________________________

______________________________________________________________________________________________

______________________________________________________________________________________________
