# Supplementary material for: Health-related quality of life among Djiboutian women with gynecological cancers, predominantly cervical cancer : a cross-sectional analytical study
Source: BMC Womens Health. 2026 Jul 31;26:561. doi: 10.1186/s12905-026-04755-x (PMC13711914; doi:10.1186/s12905-026-04755-x)
Supplement: Supplementary file 2 [file 12905_2026_4755_MOESM2_ESM.docx]

**Additional File**

**STROBE Checklist**

*Health-related quality of life among Djiboutian women with gynecological cancers, predominantly cervical cancer: a cross-sectional analytical study*

| **#** | **Section** | **STROBE item** | **Location in manuscript** |
| --- | --- | --- | --- |
| 1a | **Title/Abstract** | Indicate the study design in the title or abstract. | Title: "...a cross-sectional analytical study"; Abstract, Methods. |
| 1b | **Title/Abstract** | Provide an informative and balanced summary of what was done and found. | Structured abstract (Background/Methods/Results/Conclusion). |
| 2 | **Background** | Explain the scientific background and rationale for the study. | Introduction, §1-3. |
| 3 | **Objectives** | State specific objectives, including any pre-specified hypotheses. | Introduction, final paragraph. |
| 4 | **Methods - Design** | Present key elements of the study design. | Methods, 2.1 Study design and setting. |
| 5 | **Methods - Setting** | Describe the setting, locations, and relevant dates. | Methods, 2.1: Djibouti, 2 centres, February-April 2024. |
| 6a | **Methods - Participants** | Eligibility criteria, sources, and methods of selection. | Methods, 2.2; Figure 1 (flow diagram). |
| 6b | **Methods - Participants** | For matched studies, give matching criteria. | Not applicable (no matching). |
| 7 | **Methods - Variables** | Clearly define all outcomes, exposures, predictors, confounders, and effect modifiers. | Methods, 2.3-2.4; list of explanatory and dependent variables. |
| 8 | **Methods - Data sources** | For each variable of interest, give data sources and details of assessment/measurement methods. | Methods, 2.3-2.4; validated SF-36 + structured form (Supplementary File 1). |
| 9 | **Methods - Bias** | Describe efforts to address potential sources of bias. | Limitations (selection bias, direction of bias discussed); Methods (private interviews, single interviewer). |
| 10 | **Methods - Study size** | Explain how the study size was arrived at. | Methods; Limitations (absence of a priori sample-size calculation explicitly acknowledged). |
| 11 | **Methods - Quantitative variables** | Explain how quantitative variables were handled; what groupings were chosen and why. | Methods, 2.3-2.4 (stage, ECOG, pain groups). |
| 12a | **Methods - Statistics** | Statistical methods, including those used to control for confounding. | Methods, statistical analysis; multivariable regression. |
| 12b | **Methods - Statistics** | Methods for examining subgroups and interactions. | Not applicable (no formal interaction analysis). |
| 12c | **Methods - Statistics** | How missing data were addressed. | Methods: complete-case analysis, no imputation, with empirical verification of non-random missingness. |
| 12d | **Methods - Statistics** | If applicable, how the sampling strategy was addressed. | Not applicable (non-probabilistic consecutive sampling). |
| 12e | **Methods - Statistics** | Sensitivity analyses. | Regression diagnostics (VIF, residuals, Cook's distance) reported in the Table 4 note. |
| 13a | **Results - Participants** | Numbers of participants at each stage of the study. | Figure 1 (flow diagram: 206→90). |
| 13b | **Results - Participants** | Reasons for non-participation at each stage. | Figure 1; Methods (43 refusals, 32 unable to respond, 17 language barrier, 24 not histologically confirmed). |
| 13c | **Results - Participants** | Consider use of a flow diagram. | Figure 1. |
| 14a | **Results - Descriptive data** | Characteristics of participants and information on exposures/confounders. | Table 1. |
| 14b | **Results - Descriptive data** | Number of participants with missing data for each variable. | Methods/Results: 32 (surgery) and 29 (chemotherapy) missing; 0 for radiotherapy/hormone therapy. |
| 14c | **Results - Descriptive data** | Summarise follow-up time. | Not applicable (cross-sectional study, no longitudinal follow-up). |
| 15 | **Results - Outcome data** | Number of events or summary measures. | Table 2 (SF-36, PCS, MCS scores). |
| 16a | **Results - Main results** | Unadjusted and adjusted estimates, with precision (95% CI). | Table 3 (bivariate); Table 4 (multivariable regression with 95% CI). |
| 16b | **Results - Main results** | Categorise continuous variables where applicable. | Tables 3-4 (stage, ECOG, pain as binary categories). |
| 16c | **Results - Main results** | If applicable, translate relative risks into absolute risks. | Not applicable (linear regression, no relative risk). |
| 17 | **Results - Other analyses** | Other analyses performed (subgroups, interactions, sensitivity). | Regression diagnostics (VIF, residuals, Cook's); empirical verification of missing-data-related bias. |
| 18 | **Discussion - Key results** | Summarise key results with reference to study objectives. | Discussion, §4.1 opening. |
| 19 | **Discussion - Limitations** | Discuss limitations, sources of potential bias/imprecision, and their direction and magnitude. | Limitations (§4.6), substantially expanded: selection bias, sample size, SF-36, ethics, missing data. |
| 20 | **Discussion - Interpretation** | Give a cautious interpretation, considering objectives, limitations, multiplicity of analyses, and similar studies. | Discussion revised: "associated factors" rather than "determinants"; MCS paradox nuanced (adaptive response + statistical artefact). |
| 21 | **Discussion - Generalisability** | Discuss the generalisability (external validity) of the results. | Limitations : predominance of cervical cancer (85.6%), generalisability explicitly restricted. |
| 22 | **Other information - Funding** | Source of funding and role of funders. | Funding section (funding statement). |

**Methodological Note - SF-36 Scoring Procedure**

This note details, domain by domain, the procedure for transforming raw SF-36 items into 0-100 scores, as well as the calculation of the standardised summary scores PCS and MCS, following the method of Ware and colleagues [17,18], in response to Reviewer 3’s request for a detailed explanation of the scoring procedure.

**1. Transformation of items into domain scores (0-100)**

*For each domain, raw items are recoded according to their polarity (positive or negative), linearly transformed onto a 0-100 scale (0 = worst possible health, 100 = best possible health), then averaged within the domain.*

**Physical Functioning (PF, 10 items)**

Item score = (raw response − 1) × 50

("Yes, limited a lot" = 1 → 0; "Yes, limited a little" = 2 → 50; "No, not limited at all" = 3 → 100)

**Role-Physical (RP, 4 items, yes/no)**

Item score = (raw response − 1) × 100

(Coding: "No" = 0; "Yes" = 100, depending on the direction of the question asked)

**Bodily Pain (BP, 2 items)**

Pain intensity (6 levels): score = (6 - raw response) × 20

Pain interference (5 levels): score = (5 - raw response) × 25

BP score = average of the two transformed items

**General Health (GH, 5 items)**

Single item (general health, 5 levels): score = (5 - raw response) × 25

Items Q11a-d (true/false, 5 levels, positive or negative polarity depending on the item):

Positive items: score = (5 - raw response) × 25

Negative items: score = (raw response - 1) × 25

**Vitality (VT, 4 items) and Mental Health (MH, 5 items)**

Frequency on a 6-level scale ("All of the time" to "None of the time")

Positive items (e.g., "full of energy"): score = (6 - raw response) × 20

Negative items (e.g., "worn out"): score = (raw response - 1) × 20

**Social Functioning (SF, 2 items)**

Score = average of the two items, each transformed according to its respective scale (5 levels and 6 levels)

**Role-Emotional (RE, 3 items, yes/no)**

Same procedure as RP: item score = (raw response -1) × 100

**2. Calculation of the PCS and MCS summary scores**

The eight domain scores are standardised (transformed into z-scores) using the 1998 US general population reference norms [18], then aggregated using the published orthogonal weighting coefficients ("norm-based scoring" method), which force PCS and MCS to be statistically uncorrelated:

z_domain = (domain_score − norm_mean) / norm_SD

Raw PCS = Σ (z_domain × PCS_domain_coefficient)

Raw MCS = Σ (z_domain × MCS_domain_coefficient)

PCS = 50 + 10 × Raw PCS

MCS = 50 + 10 × Raw MCS

The weighting coefficients used are those published by Ware, Kosinski, Dewey and Gandek (2000) [18] for the 1998 US reference population, in the absence of validated coefficients specific to Djibouti or the region - a limitation explicitly acknowledged in the manuscript (Limitations section).

**3. Interpretation**

A domain score or summary score (PCS/MCS) of 50 corresponds to the mean of the 1998 US reference population, with a standard deviation of 10. A score below 50 indicates a quality of life below this reference norm; a score above 50 indicates a quality of life above this norm.

*Note: this procedure was applied identically to all study participants; it constitutes the standard published procedure for the SF-36 and does not differ according to clinical subgroup (stage, pain, etc.).*
